# Effect of inter-aural modulation depth difference on interaural time difference thresholds for speech: An observational study

**DOI:** 10.12688/f1000research.21379.1

**Published:** 2020-02-13

**Authors:** Arivudainambi Pitchaimuthu, Vibha Kanagokar, Srividya Grama Bhagavan, Jayashree S. Bhat

**Affiliations:** 1 Department of Audiology and Speech Language Pathology, Kasturba Medical College, Mangalore, Manipal Academy of Higher Education, Manipal, Mangaluru, Karnataka, 575001, India; 2 Department of Audiology, Trustwell Hospital Pvt. Limited, Bangalore, Karnataka, 560002, India

**Keywords:** Interaural time difference, modulation depth, interaural envelope difference, lateralization, localization, ITD threshold, temporal envelope

## Abstract

**Background:** The temporal envelope (ENV) plays a vital role in conveying inter-aural time difference (ITD) in many clinical populations. However, the presence of background noise and electronic features, such as compression, reduces the modulation depth of ENV to a different degree in both ears. The effect of ENV modulation depth differences between the ears on ITD thresholds is unknown; therefore, this was the aim of the current study’s investigation.

**Methods:** Six normally hearing young adults (age range 20-30 years) participated in the current study. Six vowel-consonant-vowel (VCV) (/aka/, /aga/, /apa/, /aba/, /ata/, /ada/) tokens were used as the probe stimuli. ENV depth of VCV tokens was smeared by 0%, 29%, and 50%, which results in 100%, 71%, and 50% of the original modulation depth. ITD thresholds were estimated as a function of the difference in temporal ENV depth between the ears, wherein in one ear the modulation depth was retained at 100% and in the other ear, the modulation depth was changed to 100%, 71%, and 50%.

**Results: **Repeated measures of ANOVA revealed a significant main effect of interaural modulation depth differences on the ITD threshold (F(2,10)= 9.04, p= 0.006). ITD thresholds increased with an increase in the inter-aural modulation depth difference.

**Conclusion:** Inter-aural ENV depth is critical for ITD perception.

## Introduction

The ability to locate the sound source has an important role in spatial release from masking, which results in better speech understanding in noise. When target and masker signals overlap in time but are spatially dispersed, the auditory system utilizes the spatial cues to segregate the target from the masker ^1^. Spatial acuity in the horizontal plane is essential for speech perception in noise ^2^. Inter-aural intensity differences (IID) and inter-aural time differences (ITD) due to the head shadow effect help determine the direction of the sound source in the horizontal plane. The coding of ITD and IID cues in the auditory system relies on the signal’s temporal envelope (ENV) and temporal fine structure (TFS). ENV refers to the amplitude fluctuations, and TFS refers to the fast frequency variations in the signal. ITD coding for low frequencies relies both on the TFS and ENV. However, at higher frequencies, TFS contributes mainly to IID coding, and ENV contributes predominantly to ITD coding ^3^. Hence, ITD for a speech signal is effectively conveyed through ENV, as well as the TFS, in the low frequency bands. Nevertheless, in the high frequency bands, the ITD is predominantly conveyed by the ENV.

ITD cues are more important than IID in perceiving a signal against background noise ^4^. In individuals with auditory disorders, such as cochlear hearing loss ^5^ and auditory neuropathy ^6^, the perception of TFS is affected to a greater extent than the ENV, and hence they have to rely on ITD cues present in the ENV for better localization and understanding. Even with technologically advanced hearing aids and cochlear implants, the hearing impaired still face difficulties in localization and speech understanding in noise ^7^. Band-pass filtering and compression in hearing aids and cochlear implants reduce the ENV modulation depth and onset gradient, both of which are necessary for the perception of ITD cues ^8^. ENV fluctuations will be reduced to a different degree in both the ears depending on the source location and compression setting. Other environmental factors, such as background noise and reverberation, also distort the modulation depth in both ears ^9^. However, the effect of such a difference in ENV depth between the ears on ITD thresholds is unknown. Therefore, the aim of this study was to investigate the effect of inter-aural ENV depth difference on ITD thresholds for speech stimuli.

## Methods

The study was approved (Ref No: IEC KMC MLR 12-18/506) by the Institutional Ethics Committee, Kasturba Medical College, Mangalore, India. The observational study design was used to assess the ITD thresholds of participants as a function of inter-aural ENV depth difference. All the measurements were carried out in a single session. The study was conducted at the Department of Audiology & SLP, Kasturba Medical College, Mangalore, India between 19 ^th^ December 2018 and 20 ^th^ February 2019.

### Participants

Six young adults (age range 20–30 years) participated in the perceptual experiment. The sample size was determined based on the recommendation by Anderson & Vyngris for the minimum required sample size for psychophysical research ^10^. Participants were recruited from the community through social media posts using a convenient sampling method. All participants had hearing thresholds of ≤ 15dBHL at audiometric octave frequencies. None of the participants had any history of otological and neurological disorders. Written informed consent was obtained from all participants of the study.

### Signal processing

ITD threshold for speech was measured using six vowel-consonant-vowel (VCV) (/aka/, /aga/, /apa/, /aba/, /ata/, /ada/. The VCV tokens were uttered by a female speaker, and the tokens were digitally recorded using the Praat software version 6.0.28 ^11^ installed in an HP Probook 440 G3 laptop. The tokens were acquired using an omni directional microphone connected to a high fidelity external Creative Soundblaster X-fi USB sound device. The recorded tokens were subjected to ENV modifications in the MATLAB R2017a platform ^12^ (alternatively the freely available GNU Octave, Scilab, could be used). Initially, the tokens were filtered between 80 and 7562Hz into 30 bands using third order elliptical filters. Corner frequencies of each band were determined based on the Greenwood function ^13^. Frequency bands less than 2000 Hz were discarded to avoid the contribution of TFS cues. In the remaining bands, ENV was computed as the absolute component of Hilbert transformation ^14^. The extracted ENV was low pass filtered at 128 Hz, and the depth of modulation was smeared by a factor of 0 %, 29%, and 50%, which results in 100%, 71%, and 50% of the original modulation depth. Finally, the output from the 30 bands was summed up.

### Threshold tracking procedure

ITD thresholds were estimated as a function of the difference in temporal ENV depth between the ears wherein in one ear the modulation depth was retained at 100%, and in the other ear, the modulation depth was changed to 100%, 71%, and 50% viz 100%–100%, 100%–71%, and 100%–50%. ITD thresholds were estimated separately for each of the conditions mentioned above. The stimulus presentation in one ear is delayed with reference to the stimulus presentation in the other ear. The ITD threshold is estimated as the minimum time delay required for the lateralization of the sound image. The initial presentation started with a 400μsec time delay, and the delay was adaptively varied using the transformed 2-down 1-up procedure. The time delay was increased by 25% for every negative response and decreased by 25% after two consecutive positive responses. A total of 12 reversals were administered, and midpoints of the last eight reversals were averaged to obtain the ITD threshold. Participants’ responses were acquired using a three alternative forced-choice task wherein they had to choose and indicate the sound which lateralized to the side and not the midline. For each trial, the stimulus token was randomly selected. The stimuli presentation and response acquisition was automatized using a custom-written script in MATLAB. Participants listened to the test stimuli through Sennheiser HD280 Pro headphones routed via Creative SoundBlaster X-fi USB sound device. The entire experiment was performed in a sound-treated room.

## Data analysis

The ITD threshold of each participant was estimated as the geometric average of last eight reversals of the transformed up-down procedure. A repeated measure of ANOVA was used to investigate the main effect of inter-aural modulation depth differences on ITD thresholds. The level of significance for this analysis was 0.05. A series of paired ‘t’ tests were performed for post hoc pairwise comparisons. The level of significance considered for these analyses was 0.05. However the ‘p’ level was adjusted using Holm’s sequential Bonferroni procedure ^15^ for each comparison. Statistical analyses were done using EZR version 1.35 software ^16^.

## Result and discussion

**Figure 1.**
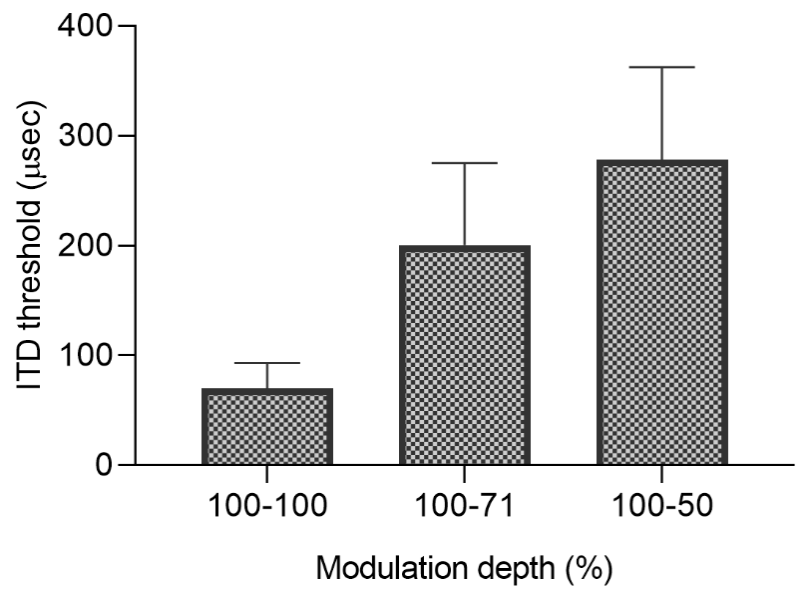
Mean and standard error of the mean of ITD thresholds for each inter-aural modulation depth condition.

Repeated measures of ANOVA revealed a significant main effect of interaural modulation depth differences on ITD threshold (F(2,10)= 9.04, p= 0.006). Post hoc pairwise comparison performed with Holm’s sequential Bonferroni correction revealed that the ITD threshold increased when the modulation depth in one ear was reduced to 71%, and this reduction was marginally significant (t= -2.37, p= 0.06). Reducing the modulation depth from 100 to 50% further increased the ITD threshold significantly (t= - 3.24, p= 0.02). ITD threshold for 50% modulation depth was significantly different from ITD threshold for 71% modulation depth (t= -5.29, p= 0.003). These results suggest that as the difference in the interaural modulation depth increases, the ITD threshold increases. The mean and standard error of the mean for each interaural modulation depth condition is represented in Figure 1.

The current study investigated the effect of inter-aural modulation depth difference on ITD thresholds. Inter-aural modulation depth differences negatively impacted ITD thresholds. ITD thresholds increased with an increase in the inter-aural modulation depth difference. Trahiotis *et al.* ^17^ indicated that the depth of amplitude modulations reaching the ears strongly influences the ITD sensitivity. The difference in the modulation depth between ears may lead to the reduction in the inter-aural ENV coherence, which would have negatively influenced the binaural processing abilities of neurons in estimating the ITD. A strong relationship between the binaural coherence and ITD cues for sound source identification has been reported in the past ^8, 18^. Results of the current study have potential implications in auditory devices such as the cochlear implant, where the ENV cues are mainly used for the perception. One limitation of the current study is the sample size. However, the small sample size could be justified as the primary aim of the current was to show the existence of an effect rather than generalizing the effect to a larger population. However, the study needs to be repeated with a large sample size to generalize the results to a large population.

## Conclusion

The temporal ENV is an essential acoustic cue for conveying sound source information, which in turn helps in source segregation. The results of this study suggest that inter-aural ENV coherence in terms of ENV depth is essential for sound source perception.

## Data availability

### Underlying data

Harvard Dataverse: ITD thresholds for temporal envelope smeared VCV tokens, https://doi.org/10.7910/DVN/OCLAE2 ^19^.

This project contains the following underlying data:

- stimuli tokens which has list of stimuli files: /aba/, /ada/, /aga/, /aka/, /apa/, /ata/.
- ITD.m and ITD_data.tal contains 2 down 1 up psychophysical procedure file
- resp.gui.fig and resp.gui.m files contain response acquisition file
- signal.speech contains signal processing and preparation of dichotic stimuli file

Data are available under the terms of the Creative Commons Zero “No rights reserved” data waiver (CC0 1.0 Public domain dedication).
