# Supplementary material for: Effect of inter-aural modulation depth difference on interaural time difference thresholds for speech: An observational study
Source: F1000Res. 2020 Feb 13;9:115. [Version 1] doi: 10.12688/f1000research.21379.1 (PMC7383721; doi:10.12688/f1000research.21379.1)
Supplement: Peer review history

# Peer review: Reviewer response for version 1

**DOI:** 10.5256/f1000research.23546.r66976

**Published:** 2020-07-24

**Authors:** James W. Dias

**Affiliations:** 1 Department of Otolaryngology, Medical University of South Carolina, Charleston, SC, USA

This study investigated whether frequency modulation depth could moderate interaural temporal difference (ITD) thresholds. As modulation depth decreased from one ear to the other, interaural time-difference thresholds increased.

 This study makes a small but meaningful contribution to the ITD literature. Though the study was conducted in a sample of normal-hearing younger adults, the results have implications for hearing replacement devices (i.e., hearing aids and cochlear implants).

 I find the methods and statistical approach to be tangible and easily understood. Though the authors address the concern in the manuscript, I am still concerned that the sample size is too small to be confident in the results. I would like to see this study replicated with a larger sample size in the future.

Is the work clearly and accurately presented and does it cite the current literature?

Partly

If applicable, is the statistical analysis and its interpretation appropriate?

Yes

Are all the source data underlying the results available to ensure full reproducibility?

Yes

Is the study design appropriate and is the work technically sound?

Yes

Are the conclusions drawn adequately supported by the results?

Partly

Are sufficient details of methods and analysis provided to allow replication by others?

Yes

Reviewer Expertise:

Audition, vision, multisensory processing, electrophysiology, speech perception

I confirm that I have read this submission and believe that I have an appropriate level of expertise to confirm that it is of an acceptable scientific standard.

# Peer review: Reviewer response for version 1

**DOI:** 10.5256/f1000research.23546.r60100

**Published:** 2020-03-19

**Authors:** M. K. Ganapathy

**Affiliations:** 1 Electrophysiology Lab, Department of Audiology, All India Institute of Speech and Hearing (AIISH), University Of Mysore, Mysore, Karnataka, India; 2 MED-EL, New Delhi, India

The study is well structured and well executed.

 Methods are appropriately framed and signal processing is well defined.  Literature indicate that ITD is drastically reduces from 1000-1500, so authors may consider filtering the signal <1500 than 2000 Hz (in case if it gives significant difference). 

 Subject size is small, however it is discussed and justified. However, in future it can be done on larger population, so the head size differences would not lead to a large SD as seen in the current study. 

 Overall well designed, with appropriate statistical analysis and well discussed.

Is the work clearly and accurately presented and does it cite the current literature?

Yes

If applicable, is the statistical analysis and its interpretation appropriate?

Yes

Are all the source data underlying the results available to ensure full reproducibility?

Yes

Is the study design appropriate and is the work technically sound?

Yes

Are the conclusions drawn adequately supported by the results?

Partly

Are sufficient details of methods and analysis provided to allow replication by others?

Yes

Reviewer Expertise:

Electrophysiology tests of hearing, pediatric audiology, cochlear implants and bone conduction implants

I confirm that I have read this submission and believe that I have an appropriate level of expertise to confirm that it is of an acceptable scientific standard.
